# Biphasic Kinetic Behavior of *E. coli* WrbA, an FMN-Dependent NAD(P)H:Quinone Oxidoreductase

**DOI:** 10.1371/journal.pone.0043902

**Published:** 2012-08-29

**Authors:** Iryna Kishko, Balasubramanian Harish, Vasilina Zayats, David Reha, Brian Tenner, Dhananjay Beri, Tobias Gustavsson, Rüdiger Ettrich, Jannette Carey

**Affiliations:** 1 Institute of Nanobiology and Structural Biology, Global Change Research Center, Academy of Sciences of the Czech Republic, Nove Hrady, Czech Republic; 2 Faculty of Sciences, University of South Bohemia, Nove Hrady, Czech Republic; 3 Chemistry Department, Princeton University, Princeton, New Jersey, United States of America; 4 Department of Biochemistry and Structural Biology, Center for Molecular Protein Science, Lund University, Lund, Sweden; Institute of Enzymology of the Hungarian Academy of Science, Hungary

## Abstract

The *E. coli* protein WrbA is an FMN-dependent NAD(P)H:quinone oxidoreductase that has been implicated in oxidative defense. Three subunits of the tetrameric enzyme contribute to each of four identical, cavernous active sites that appear to accommodate NAD(P)H or various quinones, but not simultaneously, suggesting an obligate tetramer with a ping-pong mechanism in which NAD departs before oxidized quinone binds. The present work was undertaken to evaluate these suggestions and to characterize the kinetic behavior of WrbA. Steady-state kinetics results reveal that WrbA conforms to a ping-pong mechanism with respect to the constancy of the apparent Vmax to Km ratio with substrate concentration. However, the competitive/non-competitive patterns of product inhibition, though consistent with the general class of bi-substrate reactions, do not exclude a minor contribution from additional forms of the enzyme. NMR results support the presence of additional enzyme forms. Docking and energy calculations find that electron-transfer-competent binding sites for NADH and benzoquinone present severe steric overlap, consistent with the ping-pong mechanism. Unexpectedly, plots of initial velocity as a function of either NADH or benzoquinone concentration present one or two Michaelis-Menten phases depending on the temperature at which the enzyme is held prior to assay. The effect of temperature is reversible, suggesting an intramolecular conformational process. WrbA shares these and other details of its kinetic behavior with mammalian DT-diaphorase, an FAD-dependent NAD(P)H:quinone oxidoreductase. An extensive literature review reveals several other enzymes with two-plateau kinetic plots, but in no case has a molecular explanation been elucidated. Preliminary sedimentation velocity analysis of WrbA indicates a large shift in size of the multimer with temperature, suggesting that subunit assembly coupled to substrate binding may underlie the two-plateau behavior. An additional aim of this report is to bring under wider attention the apparently widespread phenomenon of two-plateau Michaelis-Menten plots.

## Introduction

The WrbA protein from *E. coli* [1] has been identified as the founding member of a family of novel flavoproteins conserved from bacteria to higher plants [2] whose exact physiological role is still unknown [3]. Like the flavodoxins to which it is distantly related, WrbA binds FMN specifically, but with much lower affinity [4]. Its FMN-dependent NAD(P)H:quinone oxidoreductase activity [5] is presumed to be involved in quinone detoxification, consistent with available physiological evidence suggesting a role in oxidative stress defense and/or cell signaling [2], [6]. Like other quinone oxidoreductases but unlike the flavodoxins, WrbA transfers pairs of electrons without detectable formation of a stable flavin semiquinone [7]. The tetrameric WrbAs thus appear to represent a structural and functional bridge between the monomeric, FMN-dependent bacterial flavodoxins and the dimeric, FAD-dependent eukaryotic NAD(P)H:quinone oxidoreductases typified by rat NQO1, formerly called DT-diaphorase [8], which are thought to be involved in oxidative defense *via* their two-electron reduction of a wide range of quinones and other electrophiles [9]. The crystal structures of WrbAs [10] reveal cavernous active sites with a chamber above the flavin isoalloxazine ring system that appears to be big enough to accommodate either NADH as electron donor or benzoquinone (BQ; BQH2 is reduced BQ) as electron acceptor, but not both substrates simultaneously. This structural evidence suggests that the kinetic mechanism of WrbA is of the ping-pong type, as has been shown for NQO1 [11]. The present analysis was undertaken to evaluate this suggestion and to compare the steady-state kinetic properties of WrbA with the peculiar properties reported previously for NQO1.

## Results

### Michaelis-Menten Kinetics

**Figure 1:**
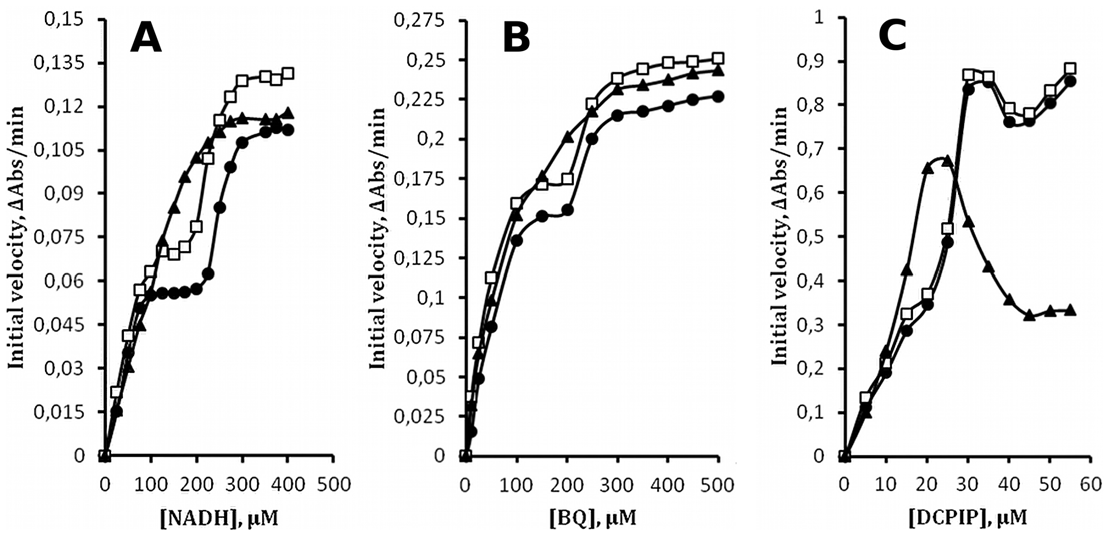
Steady-state kinetics. Initial velocity (see Methods) is plotted *vs.* substrate concentration. **A.** NADH at constant [BQ] = 50 µM. **B.** BQ at constant [NADH] = 50 µM. **C.** DCPIP at constant [NADH] = 50 µM. Each plot depicts three temperature treatments of WrbA prior to assay (see text): squares, 5°C; triangles, 23°C; circles, 5°C after 23°C. Solid lines are intended only to guide the eye and do not represent fits to the data.

Fig. 1 illustrates the Michaelis-Menten behavior of WrbA and its temperature dependence. Each panel shows representative results obtained under three conditions of enzyme preincubation, ice or 23°C for two hours, or ice °C for two hours after 23°C for two hours. Assays were conducted in a spectrophotometer at room temperature (∼23°C) for 60 sec, much shorter than the time of enzyme preincubation and too short for appreciable temperature change to occur in the reaction mixture, as determined by direct measurement.

In Fig. 1A the concentration of NADH is varied over the range 1 to 500 µM with BQ constant at 50 µM. When WrbA is handled on ice, velocity increases hyperbolically with [NADH], reaching a plateau at ∼50 µM NADH. As [NADH] increases further a second hyperbolic phase is observed beyond ∼150 µM that reaches a second plateau at ∼200 µM NADH. When WrbA is preincubated at 23°C only a single phase is observed that reaches a plateau at ∼300 µM NADH. Further incubation of the enzyme on ice after 23°C treatment results in two-plateau behavior like that observed for enzyme handled entirely on ice. The reversibility of the temperature effect suggests that the two-plateau behavior of WrbA may reflect an intramolecular conformational process. In Fig. 1B the concentration of BQ is varied over the range 1 to 500 µM with NADH constant at 50 µM. As with varying [NADH], two-plateau behavior is observed except when enzyme is preincubated at 23°C. The intermediate plateau region is detected from ∼25 to 100 µM BQ.

Because of the biphasic Michaelis-Menten behavior, these experiments were also carried out with the constant concentration of NADH or BQ set to 200 µM or 500 µM to probe both regimes (plateau regions) of the reaction. A similar temperature dependence of the two-plateau behavior is observed at these much higher concentrations as well (Figures S1 and S2).

An alternative means of monitoring the enzyme reaction was used to further examine the two-plateau behavior of WrbA. In Fig. 1C DCPIP replaces BQ, and the bleaching of its blue color at 600 nm is used to follow the time-course of reaction. Its high extinction coefficient limits its useful concentration to below ∼60 µM. At all three conditions of enzyme preincubation, as the concentration of DCPIP is varied in this range with [NADH] constant at 50 µM, an initial increase in velocity is followed by a decrease, with a velocity maximum at ∼25 to 35 µM. Except at 23°C, further increase of [DCPIP] results in increasing velocity. A second maximum is observed at ∼40 µM DCPIP when the enzyme is preincubated at 37°C (not shown). These unusual Michaelis-Menten plots with the three substrates, as well as their reversible temperature dependence, mimic surprisingly closely the kinetic behavior of the mammalian enzyme DT-diaphorase with these same substrates [11]. The presence of two plateaus in the plots implies that these proteins respond allosterically to their substrates.

### Effect of Solution Conditions

**Figure 2:**
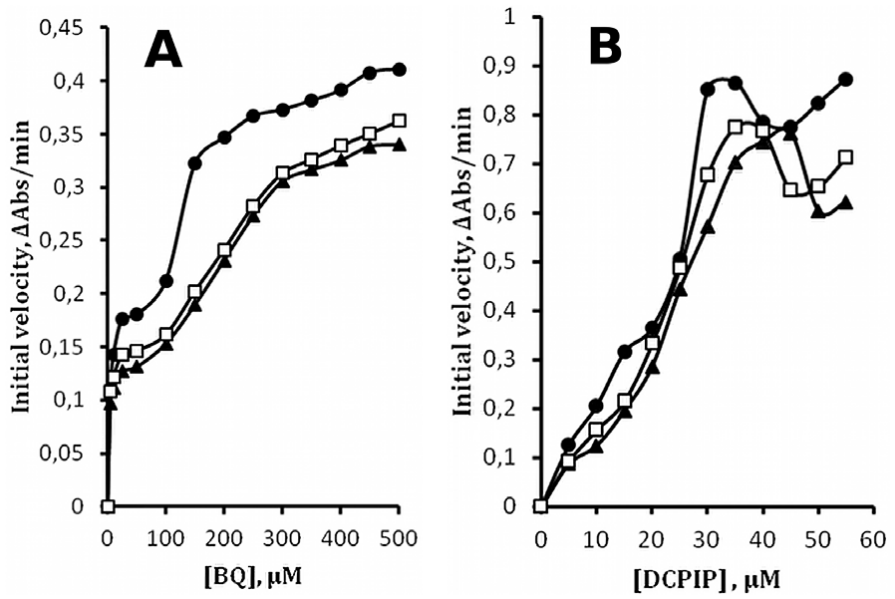
Effect of salt. Initial velocities at 5°C are plotted *vs.* substrate concentration. **A.** Titration of BQ at [NADH] = 100 µM with no salt (circles), 0.25 M NaCl (squares), and 0.5 M NaCl (triangles). **B.** Titration of DCPIP at [NADH] = 100 µM; symbols as in panel A. Solid lines are intended only to guide the eye and do not represent fits to the data.

Given the striking similarity of the two-plateau behavior of WrbA and its temperature dependence to those of DT-diaphorase, further similarities were sought in an effort to shed light on the molecular basis for these features. In the case of diaphorase the salt concentration and pH of reactions cause considerable change in the details of the two-plateau response to substrate concentration. The spectrum of conditions affecting WrbA is generally similar to those affecting diaphorase, although the detailed responses differ. For example, Fig. 2 shows the effect of salt concentration on titrations with BQ or DCPIP at constant NADH concentration when WrbA is handled on ice. The trend with increased salt concentration is that the intermediate plateau region of the BQ Michaelis-Menten plots becomes somewhat obscured, and the maxima on the DCPIP plots are shifted to higher concentrations. Diaphorase shows a different response to salt [11], with a maximum instead of an intermediate plateau in the NADH titration, and an intermediary plateau instead of a maximum in the DCPIP titration.

These very different effects of salt on the two enzymes suggest that the underlying molecular basis for the two-plateau behavior reflects specific properties of each enzyme rather than a general feature of the catalytic process. This inference implies that comparative analysis of the effects of solution conditions will probably not be informative for understanding the molecular basis of the two-plateau behavior as hoped. For this reason, pH and other solution variables known to affect diaphorase but likely to act by protein-specific mechanisms were not studied for WrbA. However, several characteristic activators and inhibitors of diaphorase enzyme activity were tested with WrbA. As for diaphorase, BSA and detergents activated WrbA, but the potent diaphorase inhibitor dicoumarol had no effect on WrbA activity (data not shown).

### Kinetic Mechanism

**Figure 3:**
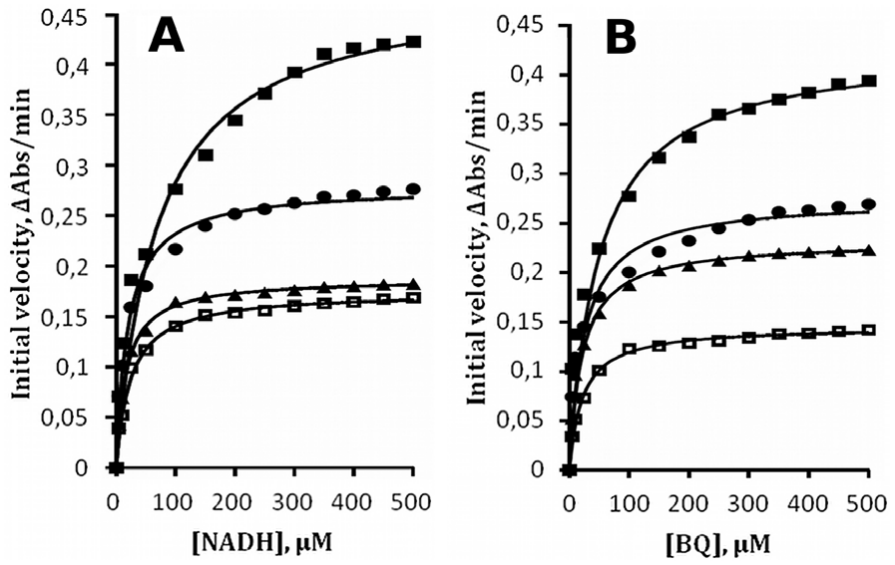
Ping-pong kinetics. Initial velocities were determined at 23°C to limit the reaction to a single kinetic phase as much as possible. **A.** Titration of NADH at [BQ] = 10 µM (open squares), 20 µM (triangles), 50 µM (circles), 100 µM (filled squares). **B.** Titration of BQ at [NADH] = 10 µM (open squares), 20 µM (triangles), 50 µM(circles), 100 µM(filled squares). Solid lines represent non-linear least-squares best fit of the Michaelis-Menten equation to the data points. The values of apparent Km and apparent Vmax returned from the fit are given in Table 1.

To evaluate the mechanism of WrbA, assays were conducted with enzyme preincubated at 23°C to limit the velocity profiles to a single hyperbolic phase. Fig. 3A shows the results of NADH titration over the range 1 to 500 µM at four fixed concentrations of BQ, 10, 20, 50, and 100 µM, and Fig. 3B shows the results of BQ titration over the range 1 to 500 uM at four fixed concentrations of NADH, 10, 20, 50, and 100 µM. In all cases approximately single-phase hyperbolas are observed, although at the higher concentrations of fixed substrate some hint of biphasic behavior is detectable. Fitting of the Michaelis-Menten equation for two substrates to each of the eight datasets by non-linear least-squares yields the apparent kinetic constants Km,app and Vmax,app, which are given in Table 1. The criterion used to establish the presence of a ping-pong kinetic mechanism is that the ratio of apparent Vmax to apparent Km be constant with substrate concentration [12]. This ratio and its standard error are given in Table 1 for each of the eight data sets. The ratio is approximately constant for the higher fixed concentrations where the velocities are most reliable, consistent with the ping-pong mechanism.

**Table 1:** Kinetic constants of WrbA^a^.

Fixed substrate (µM)	Apparent Vmax (ΔAbs/min)	Apparent Km (µM)	Vmax/Km (min^−1^ µM^−1^)	Error^b^ (min^−1^ µM^−1^)
NADH				
10	0.1421	19.0	0.00748	0.00027
20	0.2231	16.2	0.01377	0.00043
50	0.2691	20.7	0.01300	0.00048
100	0.3946	28.2	0.01377	0.00075
BQ				
10	0.1686	19.2	0.00878	0.00032
20	0.1823	14.9	0.01223	0.00035
50	0.2761	19.3	0.01431	0.00053
100	0.4224	36.3	0.01163	0.00079

a Kinetic data are presented in Figure 3.

b Errors are one standard deviation derived from triplicate measurements and propagated to the ratio Vmax/Km.

Although other categories of bi-substrate kinetic mechanism do not present constant ratios of Vmax to Km, minor deviations from constant ratios can be difficult to detect [13]. Thus the patterns of product inhibition were also analysed to enable distinction between ping-pong and random or sequential bi-bi mechanisms. Only Theorell-Chance bi-bi reaction mechanisms, in which both substrates or both products bind simultaneously but the concentration of ternary complexes is vanishingly small, cannot be distinguished from ping-pong by product inhibition, although it yields varying rather than constant ratios of Vmax to Km [12]. The product inhibition pattern expected if WrbA follows a ping-pong mechanism is that the pairs NAD/BQ and NADH/BQH2 display competitive inhibition, whereas the pairs NAD/NADH and BQ/BQH2 display non-competitive inhibition.

Table 2 summarizes the results of the eight product inhibition experiments necessary to characterize this kinetic system. Michaelis-Menten and double-reciprocal plots for these data are shown in Figure S3. The product inhibition pattern observed here is not expected for any of the bi-bi kinetic mechanisms catalogued by Cleland [12]. BQH2 shows noncompetitive inhibition in all conditions, indicating that it can bind to multiple enzyme forms or sites, even when either of the substrates (NADH or BQ) is present at high concentrations. NAD shows noncompetitive inhibition with either substrate when the other substrate is at low concentrations. At high NADH concentrations, NAD and BQ are competitive, indicating they bind to a common site or sites. At high BQ concentrations, NAD and NADH are uncompetitive indicating they bind to different forms or sites, and that at least one dinucleotide binding site does not bind BQ. Overall, the inhibition experiments indicate the presence of multiple enzyme forms or multiple sites with different affinities for the various substrates and products; kinetic results alone cannot distinguish forms from sites.

**Table 2:** Product Inhibition^a^.

Product	Varied substrate			
	NADH		BQ	
	20 µM BQ	2 mM BQ	20 µM NADH	2 mM NADH
NAD	Noncompetitive	Uncompetitive	Noncompetitive	Competitive
HQ	Noncompetitive	Noncompetitive	Noncompetitive	Noncompetitive

a Kinetic data are presented in Figure S3.

The product inhibition results therefore do not rule out unequivocally very low concentrations of ternary complexes containing both substrates or both products that would indicate a concerted reaction mechanism. The more poorly a ternary complex is populated, the smaller the deviation from constant ratios of apparent Vmax to apparent Km, and thus the more difficult to eliminate a concerted mechanism. However, the structural analysis and computational results presented below argue strongly against formation of a stable ternary complex.

### Substrate Affinity

**Figure 4:**
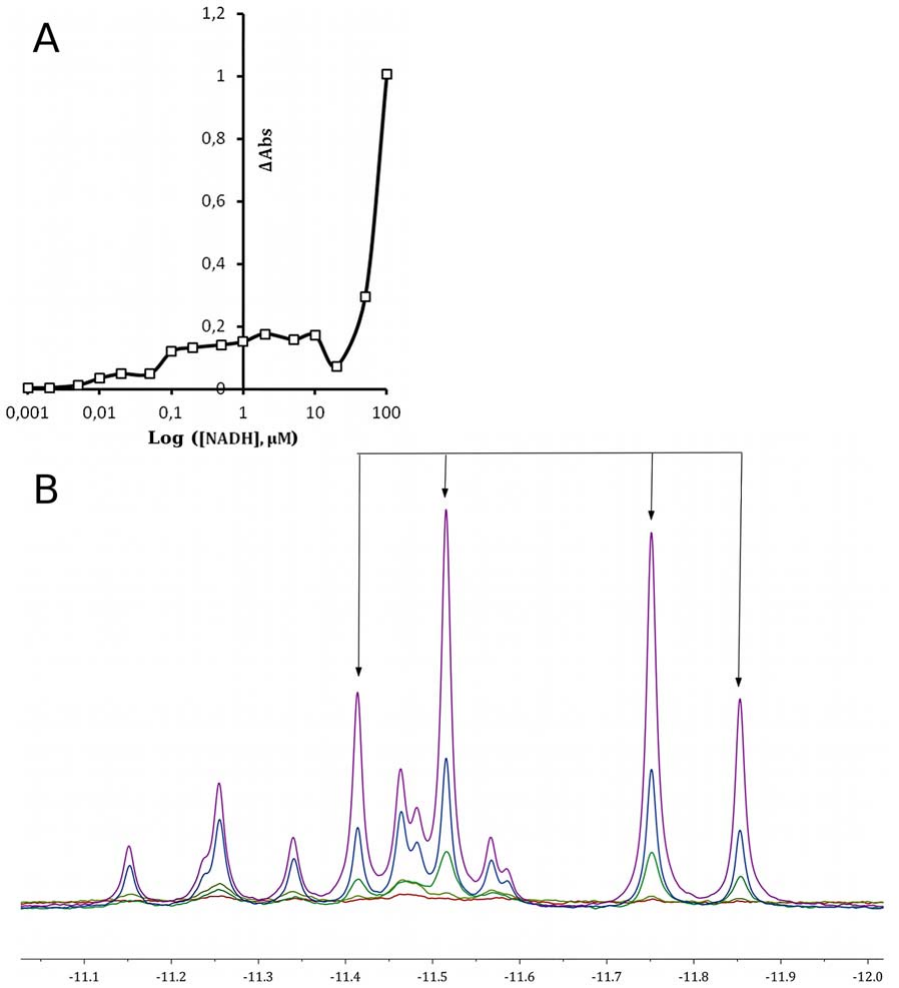
Substrate affinity. **A.** NADH binding to 50 µM apoWrbA determined by UV spectroscopy. Difference absorbance at 265 nm (see text) is plotted *vs.* [NADH]. The solid line is intended only to guide the eye and does not represent a fit to the data. **B.** NAD binding to 200 µM apoWrbA detected by ^31^P NMR. Spectra at 100, 200, 500, 1000 and 2000 µM NAD from bottom to top, respectively, are overlaid. The bracket with four arrows indicates the doublet pair characteristic of free NAD.

To evaluate whether the two-plateau Michaelis-Menten plots for WrbA reflect an underlying biphasic pattern of substrate affinity, direct studies of NADH binding affinity were attempted, first using difference absorbance spectroscopy. Because of spectral interference by the FMN cofactor these experiments could be carried out only with apoWrbA; although this form of the enzyme is not directly relevant to the Michaelis-Menten plot, if two plateaus were found with apoWrbA then a biphasic binding mechanism might also be suspected in the assay results. Absorbance spectra were acquired for protein plus and minus NADH, and the summation spectrum of protein plus substrate was subtracted from the spectrum of the mixture of protein with substrate (data not shown). From the difference spectra the wavelength 265 nm was chosen at which to report the titration of protein with NADH, as shown on Fig. 4A. The binding affinity for NADH to apoWrbA is evidently very weak. Only partial binding curves could be obtained using difference spectroscopy due to impractically high absorbance values at concentrations higher than ∼100 µM NADH, making subtraction inaccurate. The limiting value of Kd consistent with the data is ≥10–100 µM.

In an effort to determine the affinity of holoWrbA for NADH, ^31^P NMR was also used to follow the titration using the signals from the ligand, but enzymatic oxidation of NADH complicated the spectra. In the course of exploring ^31^P NMR titrations with various ligands it was observed that titration of apoWrbA with NAD yielded evidence of multiple enzyme forms or binding sites. The characteristic doublet pair of free NAD was identified by comparison with the spectrum of NAD alone. ^31^P NMR titration of apoWrbA with NAD shows that, in addition to the peaks from free NAD, eight ^31^P peaks increase in intensity as titration progresses (Fig. 4B), suggesting more than one bound form of NAD. Integration of the NMR peaks (data not shown) confirms very weak binding of NAD, precluding completion of the binding isotherm. Integration also indicates that all eight peaks accumulate in parallel at all concentrations as total NAD concentration is increased. This result allows to address whether it is multiple ligand-binding sites or multiple enzyme forms that underlie the complexities discovered in the product-inhibition experiments. Parallel accumulation is not expected for binding at distinct sites, because these are expected to have distinct affinities and thus to have non-parallel accumulation at the concentration extremes. In contrast, multiple enzyme forms would be linked to each other by mass action and hence could account for the parallel accumulation. The NMR results thus suggest that multiple forms of the apoenzyme may be reflected in the product inhibition results with holoenzyme, rather than multiple binding sites.

### Subunit Assembly

**Figure 5:**
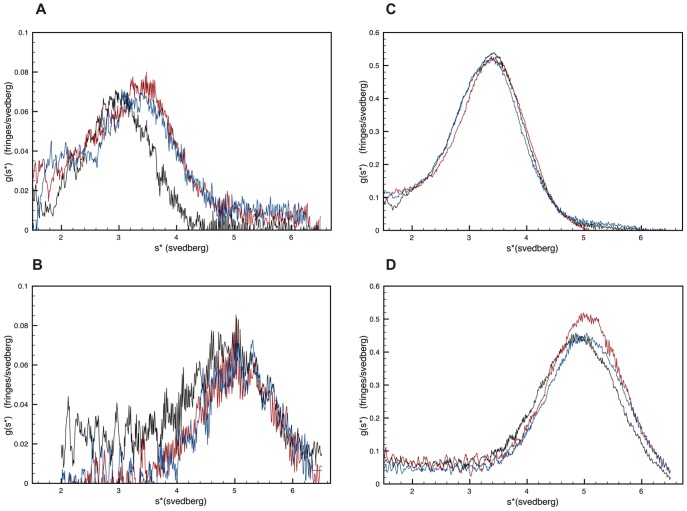
Sedimentation velocity. Each panel shows the sedimentation velocity profile using the whole boundary g(s*) approach of Stafford [28] for apoWrbA (black), WrbA+50 µM FMN (red), and WrbA+50 µM FMN+0.5 mM NAD (blue). A, 3 µM total protein (monomer) at 5°C; B, 3 µM total protein (monomer) at 20°C; C, 20 µM total protein (monomer) at 5°C; D, 20 µM total protein (monomer) at 20°C.

A dimer-tetramer assembly equilibrium has been already documented for apoWrbA by analytical ultracentrifugation (AUC) analysis [4], with a dissociation equilibrium constant of 1.4 µM (dimer), considerably above the concentration of holoWrbA used in the kinetic assays (20 nM monomer). Three of four subunits contribute residues to each active site in the WrbA tetramer [3], indicating that all four subunits are required to form the active enzyme. Thus, tetramer assembly might be related to the two-plateau kinetics. Preliminary AUC results for holoWrbA (Fig. 5) indicate a dissociation equilibrium constant at least ten times stronger than for apoWrbA, judging from the fact that apoWrbA, but not holoWrbA, displays a shift to lower S values upon dilution from 20 uM to 3 uM. (The AUC data at 3 uM are at the limit of usable signal to noise, and are shown only to enable comparison of the dilution behaviors of apo- and holoWrbA.) However, if the holoWrbA dissociation equilibrium constant were as little as ten times stronger than that of apoWrbA, then in the kinetic assays at 20 nM holoWrbA (monomer) approximately 99.9% of the enzyme would be present in the form of dimer, and only 0.1% in the form of tetramer.

Preliminary AUC results also show a large effect of temperature on the subunit assembly state of both apo- and holoWrbA, as revealed by a shift to higher S values with temperature (Fig. 5). At 5°C the mean S value for 20 µM (dimer) proteins is ∼3.6 S, whereas at 20°C the mean value is ∼5.4 S, indicating that higher temperatures favor larger assemblies. The temperature effect is seen both in presence and absence of substrate NAD and cofactor FMN, and at both high (20 µM dimer) and low (3 µM dimer) enzyme concentrations. (NADH was not used to avoid the complication of oxidation.) The predicted sedimentation coefficients calculated from the crystal structures of apo- or holoWrbA (see Methods) are 3.1 S for the dimer and 5.9 S for the tetramer. Thus, the experimental S values indicate that a mixture of dimers and tetramers is likely present in solution at both temperatures, with dimers dominating the population at low temperature and tetramers dominating at higher temperature. This finding opens the possibility that coupling of substrate binding to tetramer assembly may underlie the two-plateau kinetics. Testing this possibility will require an exhaustive series of AUC experiments at substrate concentrations corresponding to the Michaelis-Menten plots to quantify the effects on subunit assembly and their connection to the two-plateau kinetics; these experiments are beyond the scope of the present work.

### Substrate Binding Sites

Given the inconclusive product-inhibition analyses, docking analyses were used to further evaluate whether both NADH and quinone can bind simultaneously at the WrbA active site, and to calculate the free energy of binding of substrates in oxidized and reduced forms for comparison with the kinetic mechanism. These calculations require a structure with each bound substrate as a starting point. The only WrbA structure available with a bound nicotinamide dinucleotide is that of Andrade et al. (2007) [14] with PDB ID 3B6J, obtained by soaking NADH into native holoWrbA crystals. This structure revealed an unexpected position of the nicotinamide dinucleotide that the authors considered non-functional because it is too far from the flavin isoalloxazine ring for efficient electron transfer [15] (nicotinamide C4 to isoalloxazine N1, 10.4 Å; to isoalloxazine N5, 12.0 Å). The presence of a fragment of the crystallization precipitant PEG above the isoalloxazine ring in native holoWrbA crystals was presumed to preclude NADH binding in an electron-transfer-competent position [14]. The observed position lies along a hydrophobic channel that connects the active site of each WrbA monomer to the ‘poles’ of the WrbA tetramer [3]. Calculations were carried out with this structure, and docking was also used in an attempt to find an electron-transfer-competent position of the substrate. Docking analyses were combined with exact hybrid quantum mechanics/molecular mechanics (QM/MM) calculations using a specialized method that includes polarization of ligands to optimize (by re-docking) the position of substrates in the binding pocket (see Methods). In consideration of the AUC data, both the tetramer and the dimer were used in the calculations. As described in Wolfova et al. (2009) [10], the WrbA dimer is defined differently in various crystal forms; the present calculations used the dimer defined by the larger subunit interface rather than by the smaller, FMN-mediated interface.

The oxidation state of the dinucleotide in the 3B6J crystals is unclear, but if known it might shed light on whether the unusual position of that substrate represents a stage of the reaction cycle. The reported yellow color of the WrbA crystals [14] indicates the presence of oxidized FMN cofactor. Binding of NADH at the active site of oxidized holoWrbA would permit completion of the first half-reaction, the products of which are NAD and FMNH_2_. The QM/MM calculations (Table S1) indicate that NAD bound to the tetramer in the position observed in the crystal has ΔG −22.9 kcal/mol, whereas NADH in that position has ΔG 6.9 kcal/mol. The QM/MM calculations thus indicate that in the crystal position, NAD binds while NADH is repelled. These free energies of binding thus suggest that in the crystals of Andrade et al. (2007) [14] the dinucleotide has been oxidized, and its unexpected position would be consistent with a dissociation intermediate of the product NAD.

**Figure 6:**
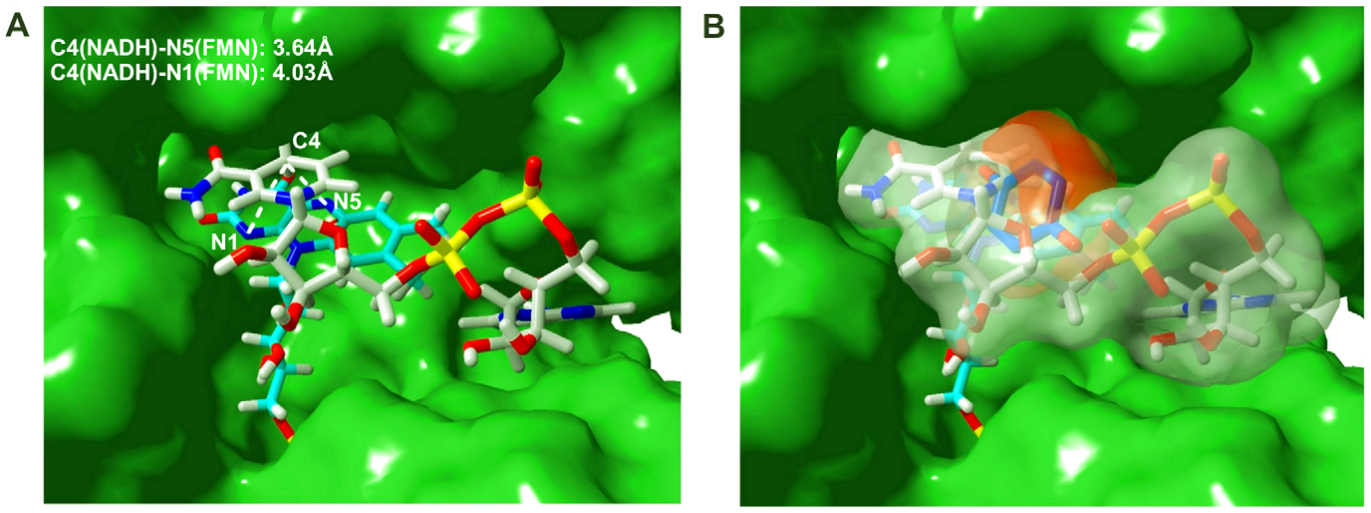
Substrate binding sites. A. NADH. View of the active site with NADH bound in the optimized position found by docking as described in the text. Green, molecular surface of holoWrbA calculated from the 2.05 Å crystal structure (PDB ID 3B6J) after removal of the FMN cofactor. Oxidized FMN is depicted as a skeletal model in atomic colors with cyan carbon, and docked NADH with white carbons for differentiation from FMN. Dashed lines represent the indicated distances in Å between nicotinamide C4 and each indicated electron acceptor site of FMN. **B. Mutual exclusivity of NADH and BQ.** Viewpoint of the binding cavity as in panel A but slightly zoomed out to better depict the steric environment of the full pocket. Translucent white indicates the molecular surface of NADH in the position identified by docking as in panel A; red indicates the molecular surface of BQ calculated from the 1.99 Å crystal structure of the BQ/WrbA complex (PDB ID 3B6K). The part of each substrate that is occluded by the other is represented by the overlap between the red and translucent white surfaces.

**Figure 7:**
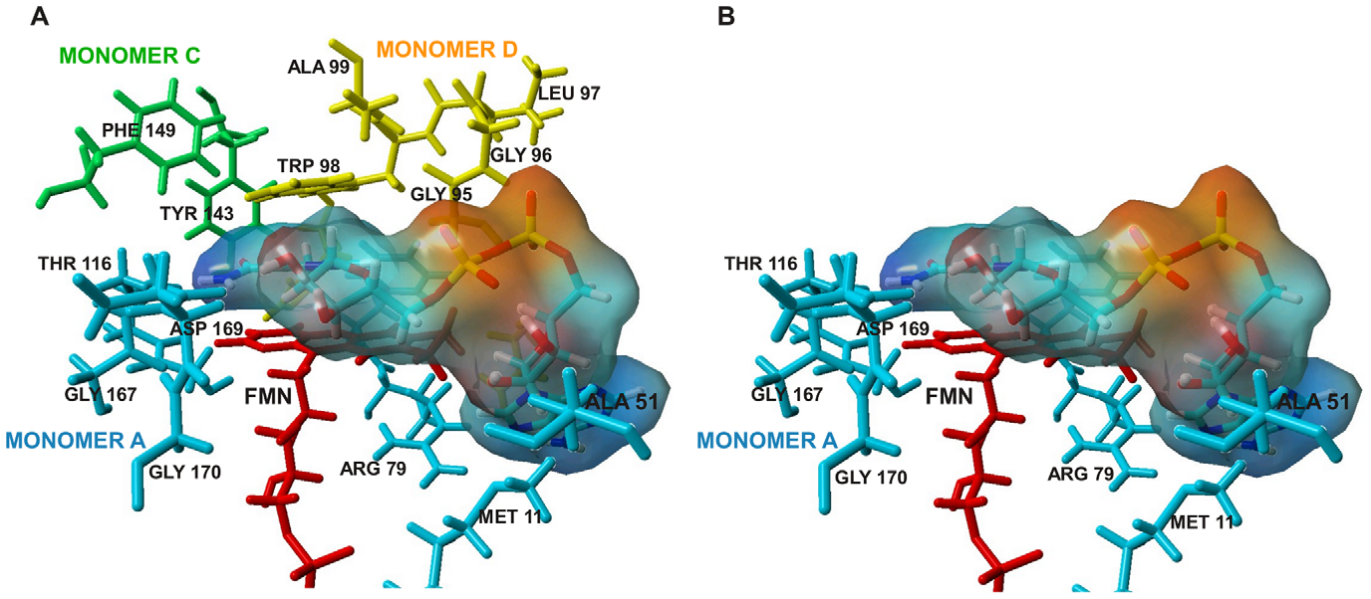
NADH contacts. View of the active site residues in direct contact with bound NADH in the optimized docked position. Oxidized FMN is depicted as a skeletal model in red; NADH is depicted as a skeletal model in atomic colors with translucent electrostatic potential surface shaded from red (negative charge) to blue (positive charge). Residues contacting NADH from monomer A are in light blue, from monomer C in green and from monomer D in yellow. **A, tetramer. B, dimer.**

The most favored binding location of NADH found by docking is in a stacked position above the isoalloxazine ring system at a distance compatible [15] with electron transfer (nicotinamide C4 to isoalloxazine N1, 4.03 Å, and to isoalloxazine N5, 3.64 Å, Fig. 6A). Residues in direct contact with bound NADH are depicted in Fig. 7. Like FMN, NADH contacts residues from three monomers in the tetramer (Fig. 7A). Seven of the NADH-contacting residues come from the monomer in which the cofactor is in proximity for electron transfer; seven other contacts come from the second dimer of the tetramer, all of which are necessarily lost upon dissociation of the tetramer into dimers (Fig. 7B). QM/MM ligand polarization calculations indicate a binding free energy for NADH of −20.2 kcal/mol in the tetramer and −18.5 kcal/mol in the dimer (Table S1). In contrast, the product NAD in the same position is repulsed in the tetramer with ΔG 9.0 kcal/mol, and bound in the dimer with ΔG −6.0 kcal/mol. The energy calculations thus suggest that in the dimeric enzyme not only is the affinity for the substrate lower, but the affinity for the product is higher. Both effects would act to reduce activity in the dimeric enzyme relative to the tetramer. The different binding free energies calculated for WrbA dimers and tetramers are consistent with the suggestion that subunit assembly may underlie its two-plateau kinetics.

The calculated free energy of binding for the second substrate, benzoquinone in the tetramer, using the structure from Andrade et al. (2007) [14] (PDB ID 3B6K), is ΔG −16.6 kcal/mol (Table S1). The main contribution to binding of benzoquinone comes from its interactions with FMNH_2_. The so-called butterfly bending of FMNH_2_ is detected in calculations on the optimized binding pocket of the reduced holoenzyme (not shown). The product hydroquinone exhibits a ΔG value of −11.2 kcal/mol, with the main binding contribution from interactions with the protein surface, and nearly negligible contribution from FMN. The calculated values indicate stronger binding of the oxidized quinone substrate than of the reduced product, consistent with the experimentally observed direction of the reaction.

Finally, the optimized, electron-transfer-competent positions of NADH and BQ derived from the computational analysis were used to further evaluate the possibility of ternary complex formation that could not be ruled out unequivocally by the product-inhibition studies. The optimized positions of the two substrates in the WrbA active site are overlaid in Fig. 6B as space-filling models. Although the two-dimensional figure is a poor representation of the volumes of the two substrates, more than 90% of the volume of bound BQ would be occluded by bound NADH. Only a small part of one edge of the quinone ring is not in steric conflict with NADH, indicating that the two substrates cannot occupy the WrbA active site simultaneously.

## Discussion

The results presented here are consistent with a kinetic mechanism of the ping-pong type for WrbA, though with at least two forms of the enzyme present as evidenced by the product inhibition and NMR results. Confirmation of a ping-pong mechanism for WrbA is consistent with the structural and computational findings that the active-site chamber, though very large, is not large enough to accommodate both NADH and BQ at the same time. The analysis presented here suggests that the non-physiological location of the nicotinamide dinucleotide in the NADH-soaked WrbA crystal [14] represents a stage of dissociation of the oxidized product NAD. The conclusion of the present work is that the most probable kinetic mechanism of WrbA is a ping-pong mechanism with multiple enzyme forms. Without extensive additional analyses the nature of these two forms can be only speculated, but an attractive possibility consistent with the preliminary AUC results presented here is that they reflect a dimer-tetramer equilibrium in the holoprotein, an hypothesis that will be evaluated by future AUC analysis.

Previous work suggested that tetrameric, FMN-dependent WrbA is a structural and functional bridge between the monomeric, FMN-dependent bacterial flavodoxins and the dimeric, FAD-dependent mammalian diaphorases [3]. A ping-pong mechanism was previously inferred for rat diaphorase [16], [17]. The two-plateau Michaelis-Menten kinetics observed for WrbA, as well as its reversible temperature dependence, are almost precisely like those reported for diaphorase [11]. The present work thus extends the similarity of WrbAs and diaphorases to include this unusual kinetic profile. Although this behavior was documented for diaphorase 40 years ago [11], [16], and was the subject of an early application of non-linear least-squares fitting of complex kinetic models to experimental data [11], the underlying molecular basis for its two plateaus has apparently never been elucidated. Substrate inhibition was offered as an early possible explanation for the two-plateau kinetic behavior of diaphorase [16], but was not considered to explain the more detailed kinetic analysis of Hollander et al. [11].

Even though a ping-pong mechanism has long been accepted for diaphorase, that enzyme also shares with WrbA some deviations that point to similar mechanistic complexities. Not all the systematic product inhibition analyses carried out for WrbA here have been reported for diaphorase, but among those that have been reported, no product inhibition is detected by NAD, and DCPIP is competitive with NADH, as is dicoumarol inhibition [16]. Both NADH and menadione are substrate inhibitors at high concentrations [16]. These deviations in the inhibition patterns for diaphorase suggest that it too may have multiple forms in solution, although diaphorase is reported to be a dimer [8] and thus may not share a subunit assembly explanation for its two-plateau kinetics.

The two-plateau results and their striking similarity to those for diaphorase prompted a literature search for other examples. Several enzymes were identified for which two-plateau kinetics have been documented (succinate dehydrogenase [18], glutamate dehydrogenase [19], cytidine triphosphate synthetase [20], phosphoenolpyruvate carboxylase [21], pyruvate kinase [22], lactate dehydrogenase [23], acetylcholinesterase [24], L-threonine dehydratase [25]). After diaphorase the most thoroughly studied with respect to its unusual kinetics is honeybee glyceraldehyde-3-phosphate dehydrogenase [26], where an intermediate plateau is present at high but not low concentrations of substrate. Several molecular explanations were considered in that case including biphasic substrate binding or subunit assembly. All were carefully ruled out, leaving no confirmed basis for the effect. Some reported examples show pH- [25] or temperature-dependent [22] interconversion between two-plateau and Michaelis-Menten behaviour, but no pattern is detectable that might lead to general insight into the phenomenon. Indeed, none of the reported examples has been plausibly explained in molecular terms. It should be noted that the reversible temperature dependence of biphasic kinetics has been documented to date only for WrbA and diaphorase. Hence, these two systems are probably the most amenable for pursuing molecular explanations, as temperature provides an easy control of the two-plateau versus Michaelis-Menten regimes. Given these numerous and long-standing examples it remains surprising that there is no confirmed molecular basis for this unusual phenomenon, nor is it widely known; given the diversity of the examples, it seems likely that the explanation may lie in a common, fundamental feature yet to be discovered.

The AUC results reported here suggest that subunit dissociation is a candidate to be that common feature in at least some cases. All the examples cited above involve enzymes that function as multimers. Enzyme dissociation has been considered to be a contributing factor to allosteric effects by Weber [27]. On the other hand, in the case of honeybee glyceraldehyde-3-phosphate dehydrogenase, subunit equilibria were ruled out on the basis of gel filtration results. Considering that interaction with the gel resin can lead to aberrations, it appears worthwhile to determine whether AUC leads to a different conclusion in that case. AUC studies under wide-ranging substrate and protein concentrations are required to evaluate the role of WrbA subunit assembly in its two-plateau kinetic behavior. Regardless of whether subunit dissociation ultimately proves to be the underlying explanation for its two-plateau kinetics, future studies of WrbA may lead to an explanation that can be evaluated for other examples as well.

The presence of two plateaus in the Michaelis-Menten plots of WrbA, diaphorase, and other cases provides *prima facie* evidence that these systems are allosteric; attempts have been made to analyze two-plateau data for other systems in light of allosteric concepts [19]. Allosteric regulation of WrbA and diaphorase implies that these enzymes are finely tuned to the physiological concentrations of their substrates, consistent with a well-defined physiological role [3] rather than the generic role in quinone detoxification that has been generally assumed.

## Methods

### Expression, Purification, and Reconstitution of WrbA

WrbA was overexpressed in BL21(λDE3) *E. coli* (Novagen) cells and purified as described previously [2] except that buffer A was 20 mM sodium phosphate pH 6.5, 1 mM EDTA, 1 mM phenylmethylsulfonyl fluoride. The pure protein was concentrated using centrifugal filter devices (Amicon) in 20 mM phosphate buffer pH 6.5. WrbA holoprotein for kinetic experiments was prepared by incubation of 20 µM apoprotein and 2 mM FMN in buffer A. Holoprotein was stored at −20°C.

### Enzyme Assay

WrbA activity was determined spectrophotometrically. Initial rates were determined by following the decrease in absorbance of NADH at 340 nm or of DCPIP at 600 nm in cuvettes of 1 cm light path. The assay solution contained 20 mM sodium phosphate, pH 6.5, 1 mM EDTA, the indicated concentrations of NADH and either DCPIP or BQ, and 20 nM WrbA in a total volume of 1 ml. The reaction was started by addition of WrbA holoprotein to the ice-cold solution with mixing by inversion; the cuvette chamber was at room temperature (∼23°C). Under these conditions the initial decrease in absorbance was linear for at least 30 seconds.

To study the influence of temperature on the steady-state kinetic behavior of WrbA, assays were done with the enzyme preincubated on ice or at room temperature (∼23°C) for two hours prior to assay. To study reversibility of the temperature effect, the protein was preincubated at room temperature for two hours, then cooled on ice for two hours prior to kinetic measurements.

Studies of kinetic mechanism used WrbA preincubated at room temperature for two hours to limit the reaction to a single kinetic phase. Kinetic data were analyzed in Excel by non-linear least-squares fitting of the Michaelis-Menten equation to yield values of apparent K_M_ and apparent V_max_. Patterns of product inhibition [12] were analyzed using NAD or BQH as product inhibitors. Each substrate (NADH, BQ) was assayed over 0 to 500 µM at 20 µM or 2 mM of the second substrate.

### Substrate Binding Affinity

UV-visible spectra were recorded from 240 to 350 nm for apoWrbA alone, substrate alone, and mixture of apoWrbA with substrate (NADH or BQ). Changes in absorbance were calculated as the difference in absorbance of the mixture minus the sum of absorbances of enzyme and substrate.

### NMR

Phosphorous-31 NMR spectra were collected at room temperature on a 500 MHz Bruker spectrometer. At each titration point, 512 scans were collected; the spectra were centered at −10 ppm with a sweep width of 50 ppm. The spectra were analyzed on MestRenova NMR software MNova with identical phasing parameters at each titration point to enable comparison of the spectra.

### AUC

Analytical ultracentrifugation experiments were performed with a Beckman-Coulter XL-I instrument at the national facility at University of Connecticut. Samples were prepared at pH 7.2 in buffer containing 20 mM phosphate and 100 mm NaCl. Data analysis used the software SEDANAL [28]. Sedimentation coefficients were calculated from the crystal structure of apoWrbA (PDB ID: 2RG1), using the program HYDROPRO [29].

### Ligand Docking and QM/MM Binding Energies

Crystal structure 3B6J [14] was used for docking of NAD and NADH. PEG fragments, AMP (adenosine monophosphate), NADH and crystal waters were removed and hydrogen atoms were added using the Maestro program from the Schrödinger software package [30]. Two systems were prepared in this way, one with FMN and NADH and another one with FMNH_2_ and NAD. The positions of the hydrogen atoms as well as the heavy atoms of the FMNH_2_ were optimized by a short steepest-descent minimization using Impact from Schrödinger [31]. Initial molecular docking of NADH or NAD to the binding pocket with the largest part formed by chain A was performed using the program Glide from the Schrödinger software package [32]. A third system was prepared leaving NADH/NAD in the position found in the crystal structure [14]. The QM/MM calculations, where NADH (NAD) was selected as the QM region and the rest of the system as MM region including implicit solvation using the Poisson-Boltzmann approach (PBS), were performed for the highest-ranked pose with a position suitable for reaction by the program Qsite from the Schrödinger package [33]. The resulting QM polarized charges of NADH (NAD) were then used for re-docking of the ligand using Glide.

Subsequent calculations of binding energies were performed for the most suitable docking poses from polarized docking as well as for the crystal structures. Calculations were also performed for the dimeric form of the protein, which was prepared by removing chains C and D in crystal structure 3B6J from the tetrameric structure. The selected structures were calculated using the QM/MM method, where NADH and FMN (or NAD and FMNH_2_) were selected as QM region and the rest of the protein as MM region. Implicit solvent was included using PBS implemented in Qsite [33]. The QM energies as well as the electrostatic contribution to QM/MM coupling energy were calculated using Gaussian 03 [34] by the density functional theory method (DFT) with a B3LYP functional including additive dispersion (treated by DFTD3 [35]) with 6–31G* basis set. The basis set superposition error was treated by the counterpoise correction method [36]. Van der Waals contribution to the QM/MM coupling energy was calculated by the MM program Impact [32] using the OPLS2005 forcefield [37]. The same calculations were also performed using the crystal structure 3B6 K [14] containing benzoquinone (hydroquinone) as ligand.

## Supporting Information

Figure S1 **Kinetics of WrbA at high concentrations of NADH or BQ.** Assays were carried out at 5°C to reveal the two-plateau behavior.
(PDF)

Figure S2 **Kinetics of WrbA at high concentrations of NADH or BQ.** Assays were carried out at 23°C to limit the reaction to a single kinetic phase.
(PDF)

Figure S3 **Product inhibition of WrbA.** Each pair of plots shows rectangular hyperbolic and linear representations of the same data set for the indicated concentrations of constant and variable substrates and inhibitors.
(PDF)

Table S1 **Ligand binding energies calculated by QM/MM method.**
(PDF)
