# Supplementary material for: Biphasic Kinetic Behavior of E. coli WrbA, an FMN-Dependent NAD(P)H:Quinone Oxidoreductase
Source: PLoS One. 2012 Aug 29;7(8):e43902. doi: 10.1371/journal.pone.0043902 (PMC3430622; doi:10.1371/journal.pone.0043902)
Supplement: Figure S1 — Kinetics of WrbA at high concentrations of NADH or BQ. Assays were carried out at 5°C to reveal the two-plateau behavior. (PDF) [file pone.0043902.s001.pdf]

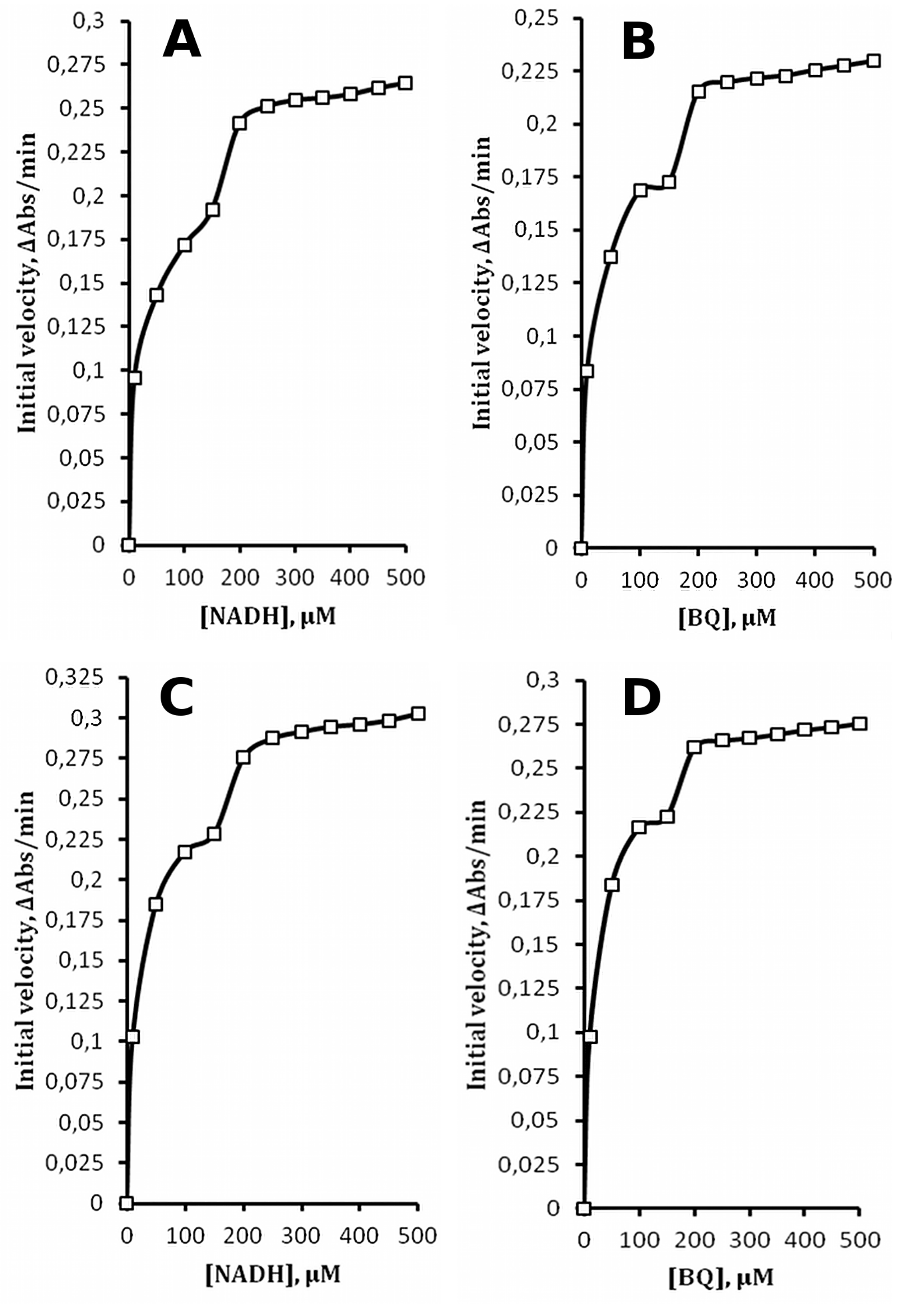

Figure S1. Kinetics of WrbA at high concentrations of NADH or BQ. Assays were carried out at 5 °C to reveal the two-plateau behavior.
