# Supplementary material for: Biphasic Kinetic Behavior of E. coli WrbA, an FMN-Dependent NAD(P)H:Quinone Oxidoreductase
Source: PLoS One. 2012 Aug 29;7(8):e43902. doi: 10.1371/journal.pone.0043902 (PMC3430622; doi:10.1371/journal.pone.0043902)
Supplement: Figure S2 — Kinetics of WrbA at high concentrations of NADH or BQ. Assays were carried out at 23°C to limit the reaction to a single kinetic phase. (PDF) [file pone.0043902.s002.pdf]

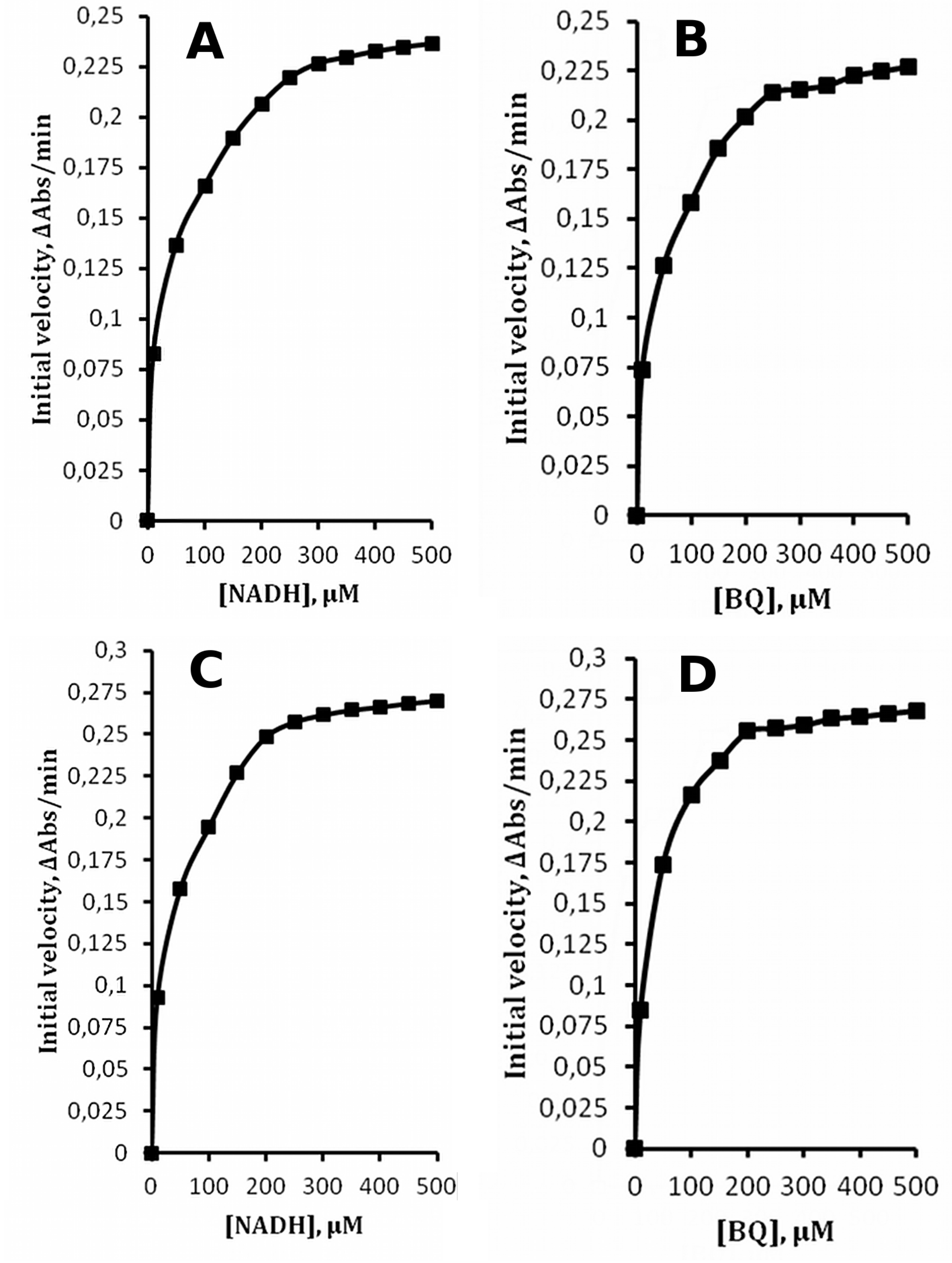

Figure S2. Kinetics of WrbA at high concentrations of NADH or BQ. Assays were carried out at 23 °C to limit the reaction to a single kinetic phase.
