# Supplementary material for: Biphasic Kinetic Behavior of E. coli WrbA, an FMN-Dependent NAD(P)H:Quinone Oxidoreductase
Source: PLoS One. 2012 Aug 29;7(8):e43902. doi: 10.1371/journal.pone.0043902 (PMC3430622; doi:10.1371/journal.pone.0043902)
Supplement: Figure S3 — Product inhibition of WrbA. Each pair of plots shows rectangular hyperbolic and linear representations of the same data set for the indicated concentrations of constant and variable substrates and inhibitors. (PDF) [file pone.0043902.s003.pdf]

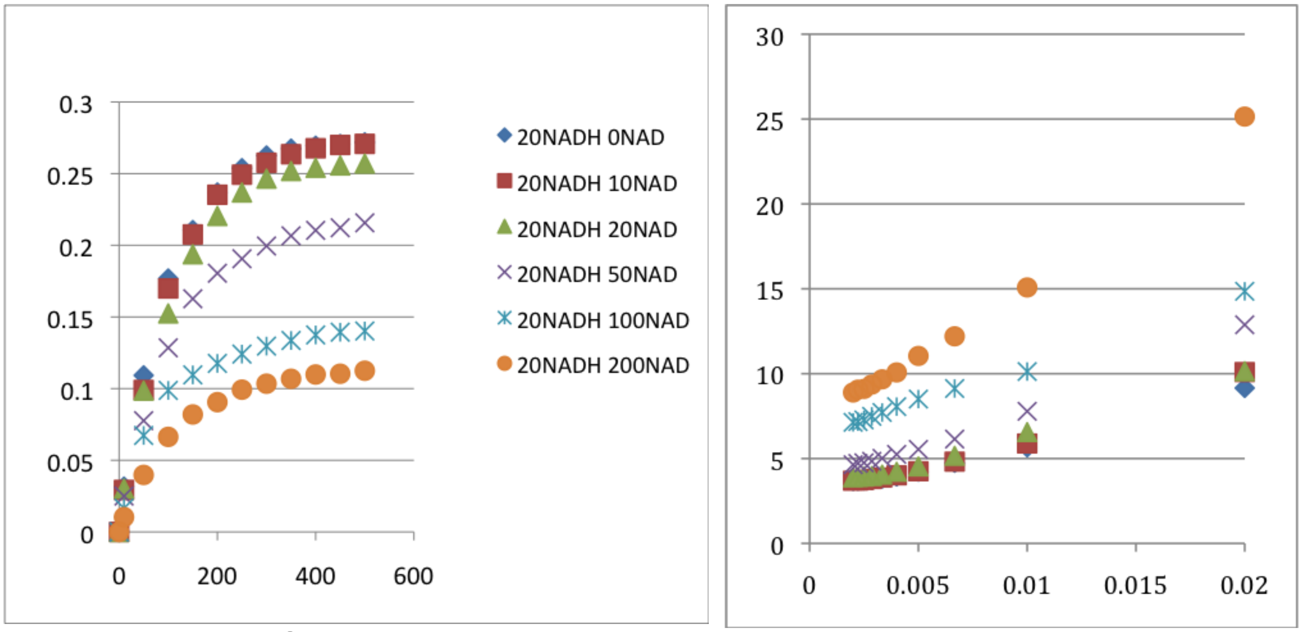

A. NAD vs BQ at unsaturated NADH

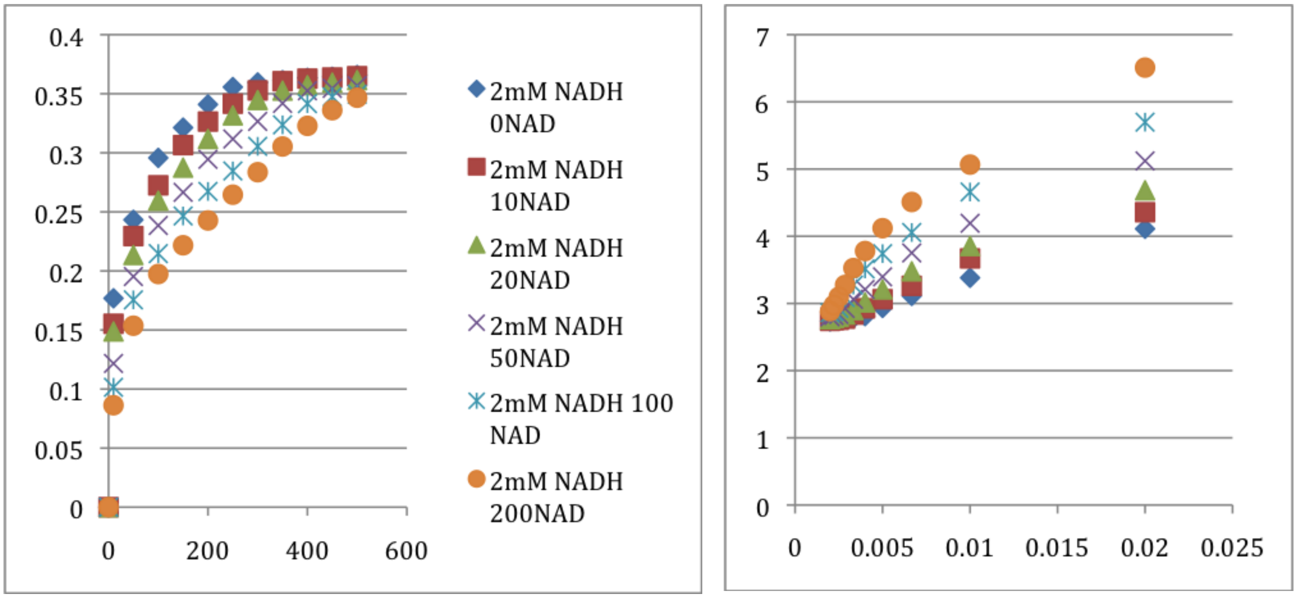

B. NAD vs BQ at saturating NADH

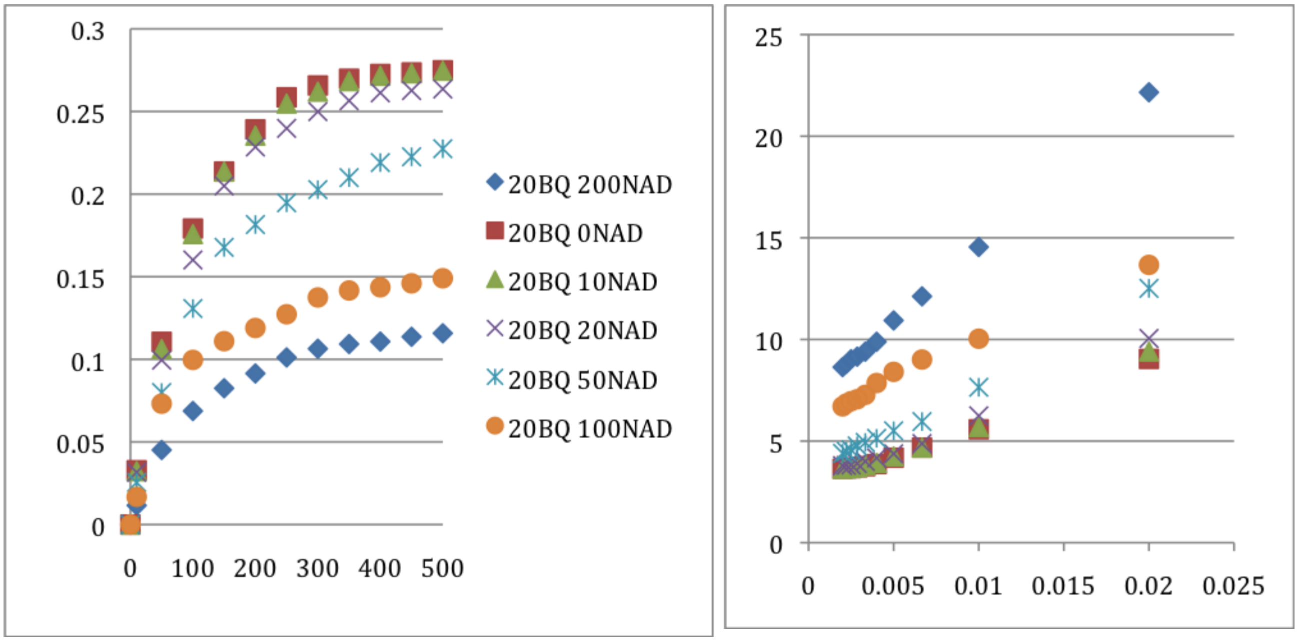

C. NAD vs NADH at unsaturating BQ

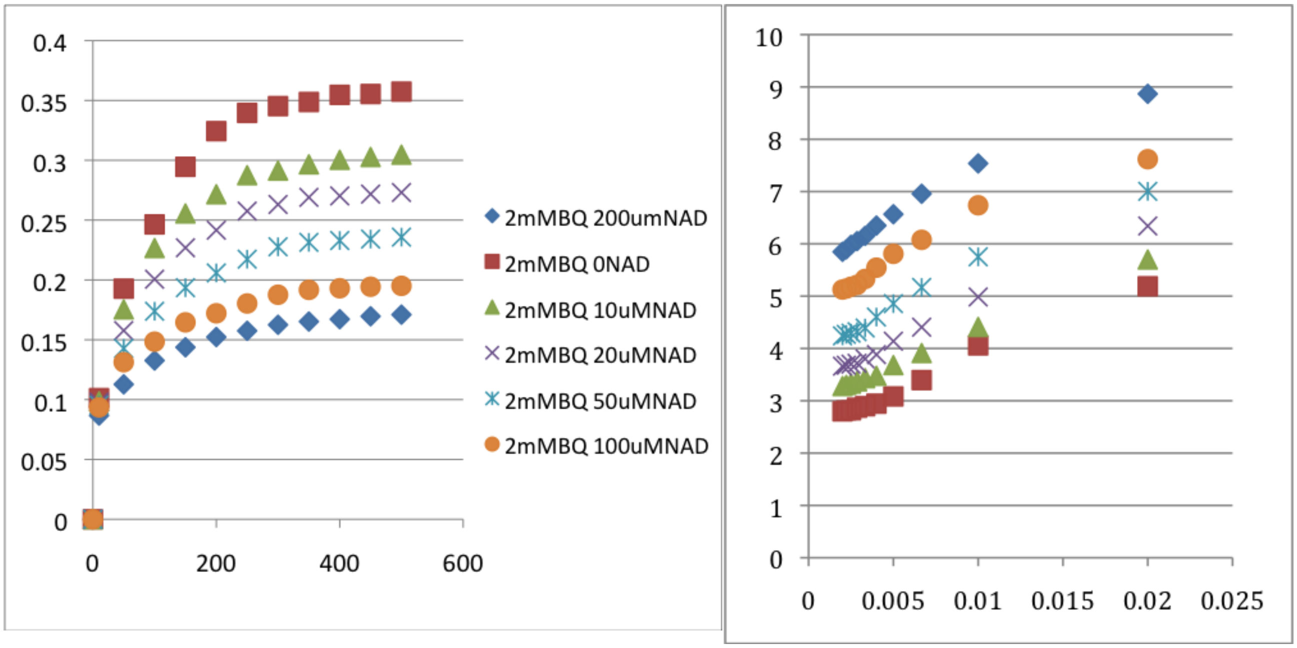

NAD vs NADH at saturating BQ

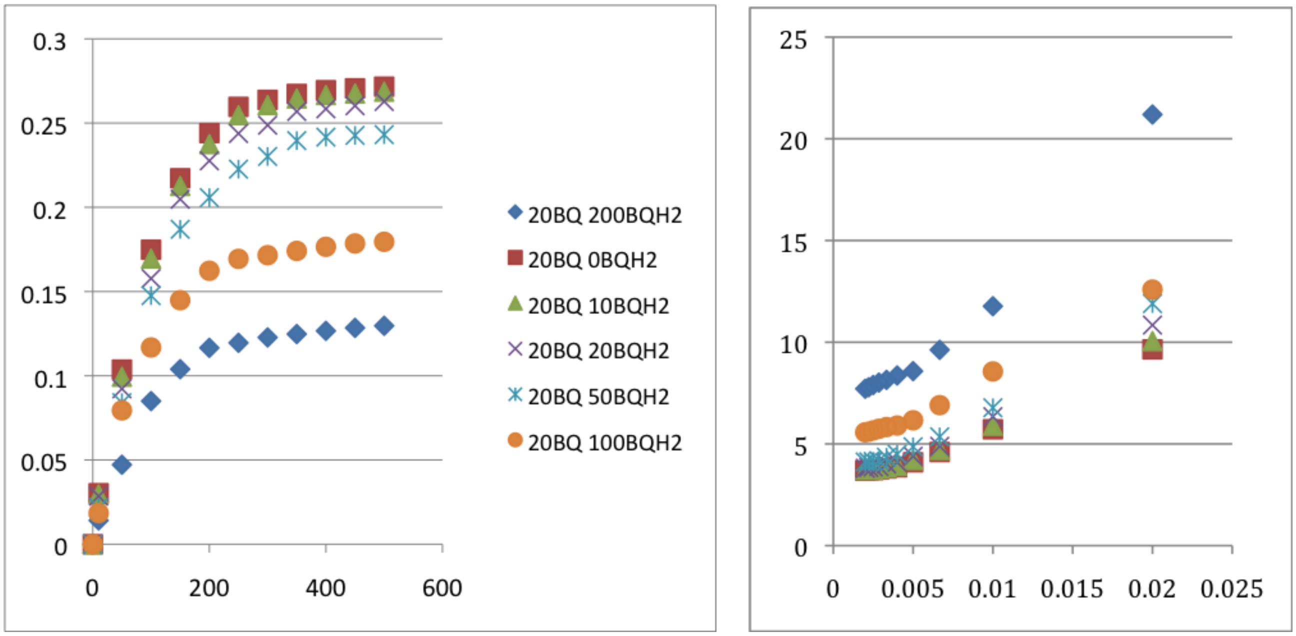

E. BQH2 vs NADH at unsaturating BQ

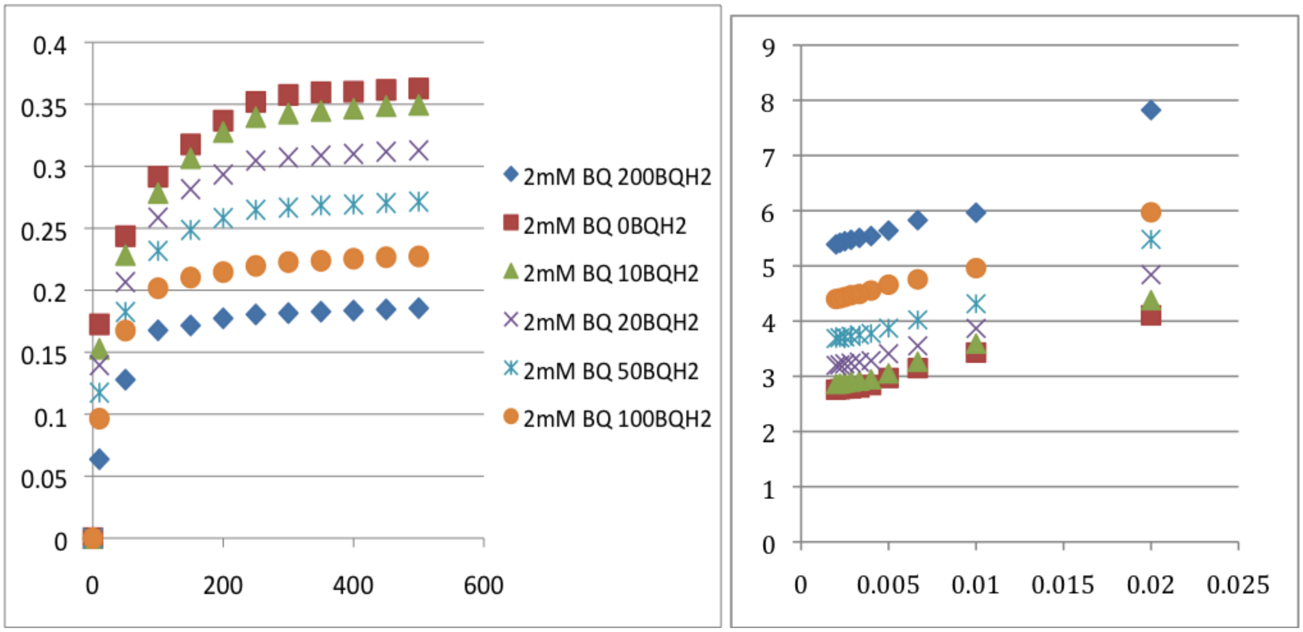

F. BQH2 vs NADH at saturating BQ

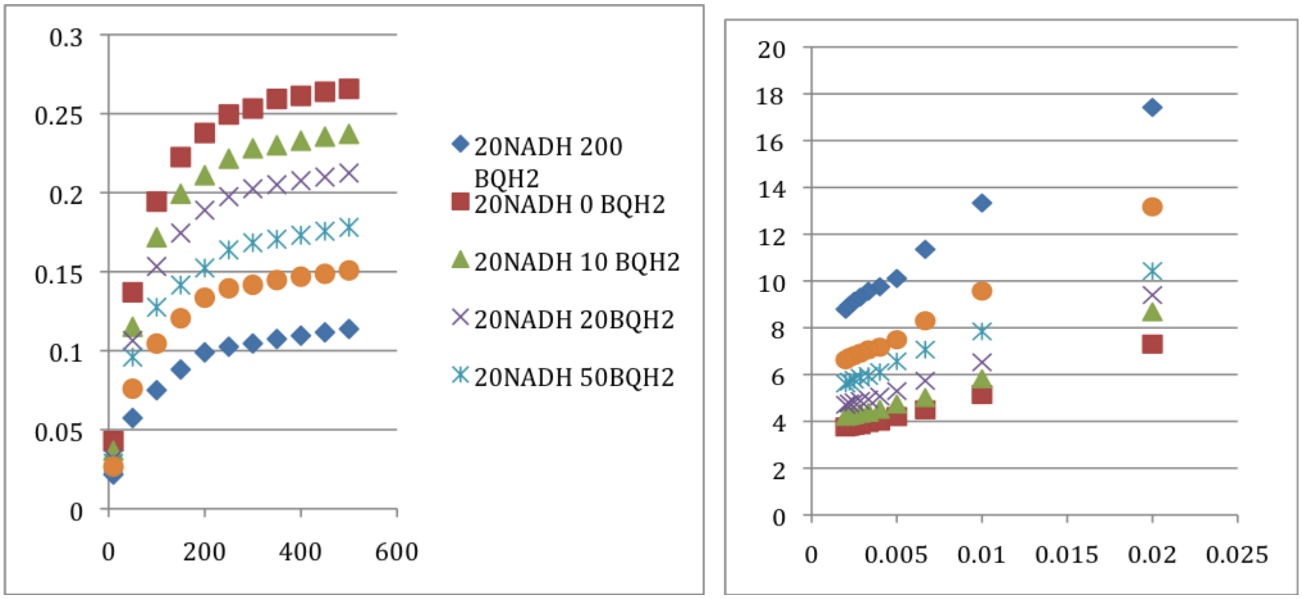

G. BQH2 vs BQ at unsaturating NADH

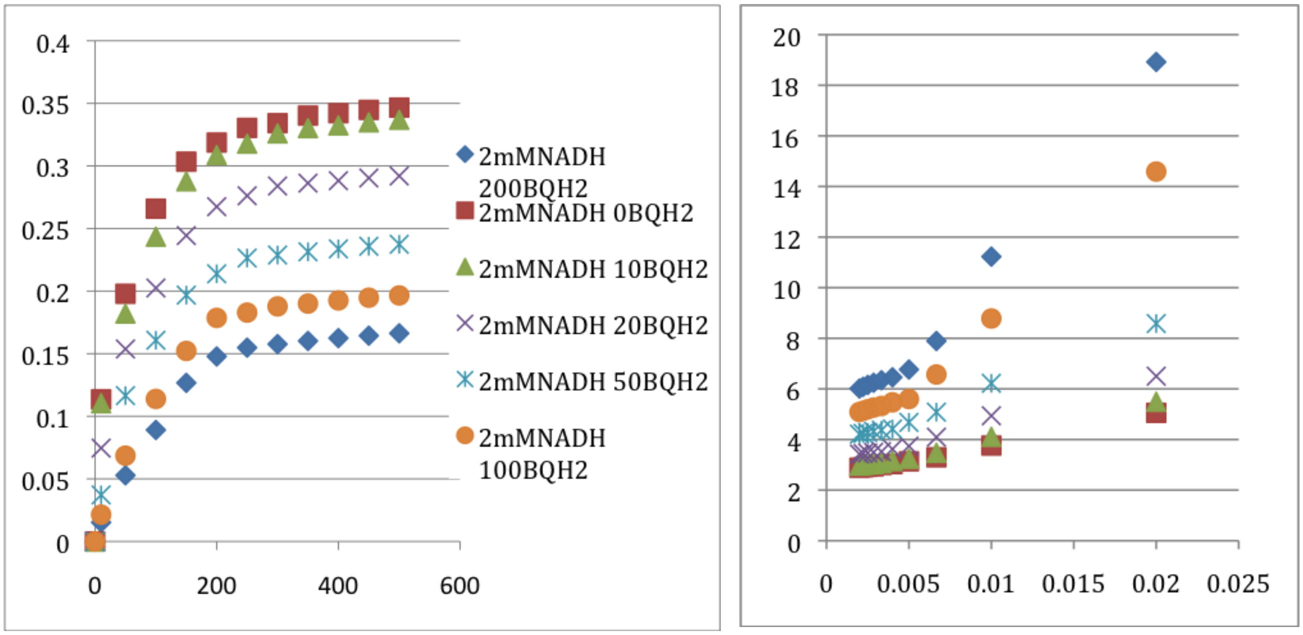

H. BQH2 vs BQ at saturating NADH

Figure S3. Product inhibition of WrbA. Each pair of plots shows rectangular hyperbolic and linear representations of the same data set for the indicated concentrations of constant and variable substrates and inhibitors.
