# Supplementary material for: Biphasic Kinetic Behavior of E. coli WrbA, an FMN-Dependent NAD(P)H:Quinone Oxidoreductase
Source: PLoS One. 2012 Aug 29;7(8):e43902. doi: 10.1371/journal.pone.0043902 (PMC3430622; doi:10.1371/journal.pone.0043902)
Supplement: Table S1 — Ligand binding energies calculated by QM/MM method. (PDF) [file pone.0043902.s004.pdf]

Table S1: Ligand binding energies calculated by QM/MM method[a]

	QM[b]	QM/MM[b]	SOLV[b]	**Tot[b]**
**Docked to reactive position**	kcal/mol	kcal/mol	kcal/mol	**kcal/mol**
Tetramer+FMN+NADH	92.26	281.98	-394.39	**-20.15**
Tetramer+FMNH2+NAD	-14.39	159.83	-136.43	**9.01**
Dimer+FMN+NADH	92.26	13.20	-123.96	**-18.50**
Dimer+FMNH2+NAD	-14.39	47.65	-39.23	**-5.97**
Tetramer+FMNH2+benQ	-45.69	21.81	7.27	**-16.62**
Tetramer+FMN+hydQ	-5.77	-23.81	18.34	**-11.23**
**Position in crystal structure**				
Tetramer+FMN+NADH	113.00	256.09	-362.16	**6.93**
Tetramer+FMNH2+NAD	14.51	113.65	-151.06	**-22.90**
Tetramer+FMNH2+benQ	-53.93	40.34	6.46	**-7.13**
Tetramer+FMN+hydQ	-3.44	-14.96	11.20	**-7.2**

[a]Ligand binding energies of the reactants (NADH, benQ=benzoquinone) and products (NAD^+, hydQ=hydroquinone) to the complex of WrbA with cofactor FMN or $FMNH_2$ calculated by QM/MM method.

[b]The components of the QM/MM binding energy: **QM**, interaction energy within QM region (between ligand and FMN); **QM/MM**, interaction energy between QM region (ligand+FMN) and MM region (protein); **SOLV**, solvation energy; **Tot**, total binding energy.
